# Adjuvant interferon in melanoma – a resurrection?

**DOI:** 10.1054/bjoc.2001.1798

**Published:** 2001-05

**Authors:** M R Middleton, N Thatcher

**Affiliations:** CRC Department of Medical Oncology, Christie Hospital, Manchester M20 9BX, UK


*British Journal of Cancer* (2001) **84**(9), 1141–1142
© 2001 Cancer Research Campaign
doi: 10.1054/ bjoc.2001.1798, available online at http://www.idealibrary.com on IDEAL®

http://www.bjcancer.com

Editorial

### Adjuvant interferon in melanoma – a resurrection?

**MR Middleton and N Thatcher**

CRC Department of Medical Oncology, Christie Hospital, Manchester M20 9BX, UK

Results of the Eastern Co-operative Oncology Group (ECOG) 1684 trial of high-dose interferon (HDI) versus observation in resected stage IIB or III melanoma (primary tumour >4 mm in depth and/or lymph node involvement) were published in 1996 (Kirkwood et al, 1996). The study was the first to show a statistically significant improvement in relapse-free and overall survival in these patients with a high risk of relapse. This was hailed as a breakthrough in the USA: here at last, after many negative studies, was an effective treatment. In Europe the response was more muted, with many oncologists unwilling to submit patients to such an arduous regimen without further supportive evidence. The results of the Intergroup E1690 study were therefore disappointing: although the improvement in relapse-free survival with high-dose treatment seen in the ECOG 1684 trial was confirmed there was no improvement in overall survival (Kirkwood et al, 2000a).

The difference in these results is due to an improvement in the outcome of patients kept under observation in the later trial, there being an increase in median survival after relapse from 1.8 years in ECOG 1684 to 4.3 years in E1690. The reasons for this remain unclear: salvage therapy with interferon at relapse accounts for some of the increase and there were differences between the two study populations. Whatever the explanation, these conflicting reports on the efficacy of HDI have led to only limited uptake of the regimen in the UK to date.

A number of studies of low-dose interferon (LDI) in this indication have also been undertaken. The first major study, conducted under the auspices of the World Health Organization, showed no improvement in relapse-free or overall survival for patients with stage III disease at long-term follow-up (Cascinelli, 1999), a finding in common with the LDI arm of E1690. Two large trials have examined LDI treatment in patients with resected stage II disease, i.e. tumours >1.5 mm in depth without nodal metastases. An Austrian study of over 300 patients demonstrated increased disease-free survival for a year of LDI, compared with observation, but as yet no effect on overall survival (Pehamberger et al, 1998). The larger French study compared 18 months LDI with observation in 489 patients. Again, there was a clear advantage for LDI in terms of disease-free survival, and a trend towards improved overall survival (Grob et al, 1998). Similar results with only 6 months treatment are published in this issue by the Scottish Melanoma Group, although the relatively small numbers recruited to their study preclude meaningful statistical analysis (Cameron et al, 2001). The UKCCCR AIMHIGH study, which has recently closed to recruitment, will provide more evidence on the effects of LDI in the stage IIB and III population.

Results from the latest Intergroup trial, E1694, are now available. This study randomized patients between HDI (according to the regimen in the earlier ECOG studies) and a GM2 ganglioside vaccine. The study was designed before the results of E1690 were known, to assess whether the putative benefits of HDI could be equalled or exceeded by the less toxic vaccine regimen. The trial was closed by the National Cancer Institute's Drug Data Safety Monitoring Committee due to evidence for the clear superiority of HDI over GM2 vaccination. E1694 recruited 880 patients with resected stage IIB and III melanoma, stratified for the number of nodes involved. The planned interim analysis that resulted in study closure examined the outcome in 774 eligible patients. HDI-treated patients had improved relapse-free (hazard ratio 1.47; $P = 0.0015$) and overall survival (hazard ratio 1.52; $P = 0.009$) compared with those vaccinated (Kirkwood et al, 2000b).

Can HDI now be considered as the treatment of choice for melanoma at high risk of recurrence? Unless one postulates a detriment from GM2 vaccination the results of this latest, and largest, Intergroup study are compelling. Earlier trials with the vaccine showed no such detriment (Livingston et al, 1994), there were no treatment-related deaths in the phase III trial and mounting an antibody response to the vaccine did not affect survival. Furthermore, the survival curve for vaccinated patients in E1694 tracks that of the observation arm of E1690.

HDI has a place in the management of melanoma, but questions remain as to the duration and dose required and the population who should receive treatment. The lack of understanding of the basis for interferon's activity has hampered the search for answers to these questions. Nevertheless valuable insights can be gained from the results of clinical trials published to date. Separation of the survival curves was seen very early in the course of E1684, and the differences noted in E1694 are apparent after less than 18 months' follow up. The early, intravenous, component of the HDI regimen appears important for its effect on melanoma, and it is unclear what role subsequent intermediate-dose interferon administration plays. The E1697 trial of the intravenous month of HDI versus observation in resected T3N0 melanoma may help to clarify the issue. The imminent results from the EORTC 18952 study will elucidate the role for intermediate doses of interferon as adjuvant therapy for melanoma. Given the costs and toxicities of HDI narrower identification of patients who stand to gain from treatment would be advantageous. The role of established and novel staging investigations in selecting patients needs to be defined, and surrogate markers for response identified. However, none of the studies above was powered to address which sub-populations most benefit from treatment. It should be noted that the benefits of HDI identified in the Intergroup studies were broadly similar in all of the sub-groups making up the high-risk melanoma population.

The results of E1694 establish HDI as the benchmark adjuvant therapy for resected stage IIB and III melanoma. However, questions remain as to whether the full ECOG regimen is needed and more precise identification of the population that should be treated is required in order to offset the acknowledged toxicity of HDI.

For patients with stage IIA disease there is, at present, no treatment shown to improve overall survival although ample evidence exists for a prolongation of relapse-free survival with LDI.

### REFERENCES

Cameron DA, Cornbleet MC, Mackie RM, Hunter JAA, Gore M, Hancock B and Smyth J (2001) Adjuvant interferon alpha 2b in high risk melanoma – the Scottish study. *Br J Cancer* **00**: 000–000

Cascinelli N (1999) Data presented at the 3rd International Conference on Adjuvant Therapy of Malignant Melanoma. London

Grob JJ, Dreno B, de la Salmoniere P, Delauney M, Cupissol D, Guillot B, Souteyrand P, Sassolas B, Cesarini JP, Lionnet SL, Chastang C and Bonerandi JJ (1998) Randomised trial of interferon alpha-2a as adjuvant therapy in resected primary melanoma thicker than 1.5 mm without clinically detectable node metastases. French Co-operative Group on Melanoma. *Lancet* **351**: 1905–1910

Kirkwood JM, Strawderman MH, Ernstoff MS, Smith TJ, Bordern EC and Blum RH (1996) Interferon alfa-2b adjuvant therapy of high-risk resected cutaneous melanoma: the Eastern Co-operative Oncology Group trial EST 1684. *J Clin Oncol* **14**(1): 7–17

Kirkwood JM, Ibrahim JG, Sondak VK, Richards J, Flaherty LE, Ernstoff MS, Smith TJ, Rao U, Steele M and Blum RH (2000a) High- and low-dose interferon alfa-2b in high-risk melanoma: first analysis of Intergroup trial E1690/S9111/C9190. *J Clin Oncol* **18**: 2444–2458

Kirkwood JM, Ibrahim JG, Sondak VK, Sosman JA and Ernstoff MS (2000b) Relapse-free and overall survival are significantly prolonged by high-dose IFNα2b (HDI) compared to vaccine GM2-KLH with QS21 (GMK, Progenics) for high-risk resected stage IIB-III melanoma: results of the intergroup phase III study E1694/S9512/C509801. *Eur J Cancer* **11**(4): abstract 40

Livingston P, Wong GYC, Adluri S, Padavan M, Parente R, Hanlon C, Calves MJ, Helling F, Ritter G, Oetgen HF and Old LJ (1994) Improved survival in stage III melanoma patients with GM2 antibodies: a randomized trial of adjuvant vaccination with GM2 ganglioside. *J Clin Oncol* **12**: 1036–1044

Pehamberger H, Soyer HP, Steiner A, Kofler R, Binder M, Mischer P, Pachinger W, Aubock J, Fritsch P, Kerl H and Wolff K (1998) Adjuvant interferon alfa-2a in resected primary stage II cutaneous melanoma. *J Clin Oncol* **16**: 1425–1429

*© 2001 Cancer Research Campaign*

